# Synthesis and Characterization of Superhydrophobic, Self-cleaning NIR-reflective Silica Nanoparticles

**DOI:** 10.1038/srep35993

**Published:** 2016-11-08

**Authors:** Deepa Sriramulu, Ella Louise Reed, Meenakshi Annamalai, Thirumalai Venky Venkatesan, Suresh Valiyaveettil

**Affiliations:** 1 Department of Chemistry, 3 Science Drive 3, National University of Singapore (NUS), 117543, Singapore; 2 NUSNNI-NanoCore, National University of Singapore, 117411, Singapore; 3 Department of Physics, National University of Singapore, 117542, Singapore; 4 Department of Electrical and Computer Engineering, National University of Singapore, 117576, Singapore; 5 Department of Material Science and Engineering, National University of Singapore, 117575, Singapore; 6 NUS Graduate School for Integrative Sciences & Engineering, National University of Singapore, 117456, Singapore

## Abstract

Multifunctional coatings offer many advantages towards protecting various surfaces. Here we apply aggregation induced segregation of perylene diimide (PDI) to control the surface morphology and properties of silica nanoparticles. Differentially functionalized PDI was incorporated on the surface of silica nanoparticles through Si-O-Si bonds. The absorption and emission spectra of the resultant functionalised nanoparticles showed monomeric or excimeric peaks based on the amounts of perylene molecules present on the surface of silica nanoparticles. Contact angle measurements on thin films prepared from nanoparticles showed that unfunctionalised nanoparticles were superhydrophilic with a contact angle (CA) of 0°, whereas perylene functionalised silica particles were hydrophobic (CA > 130°) and nanoparticles functionalised with PDI and trimethoxy(octadecyl)silane (TMODS) in an equimolar ratio were superhydrophobic with static CA > 150° and sliding angle (SA) < 10°. In addition, the near infrared (NIR) reflectance properties of PDI incorporated silica nanoparticles can be used to protect various heat sensitive substrates. The concept developed in this paper offers a unique combination of super hydrophobicity, interesting optical properties and NIR reflectance in nanosilica, which could be used for interesting applications such as surface coatings with self-cleaning and NIR reflection properties.

Silica nanoparticles are used in medical, biological and chemical industries owing to their low toxicity, chemical inertness, optical transparency and biocompatibility^1^,^2^. Dye-doped silica nanoparticles are designed and tested for potential uses in biomedical and coating applications^3^,^4^ due to their highly desirable properties that include excellent photostability, tunable optical properties, controllable size, thermal and environmental stability and a large surface area to volume ratio^5^.

Perylene derivatives have been identified as fluorophores with high fluorescence quantum yield, good photostability^6^ and a high molar extinction coefficient (10^5 ^M^−1 ^cm^−1^ for isolated perylene bisimide chromophores)^7^ owing to a planar and highly conjugated structures. Furthermore, the high reflectance in the NIR region of the electromagnetic spectrum allows the incorporation of thermal insulating properties into materials^8^. Organic NIR absorbers have a wide range of applications such as invisible inks, NIR readable bar codes, laser-welding of plastics, laser induced thermal curing^9^, and energy saving applications^10^. The origin of the NIR reflectance is due to the high refractive index of the perylene derivatives incorporated on various coatings^8^,^10^. NIR fluorescent nanoparticles are useful as they provide negligible background fluorescence, low-inner-filtration interference in complex biological systems such as whole blood^11^ and used as substrates for the characterization of enzymatic processes^12^.

The observed hydrophobicity of the lotus leaf surface has led to extensive research into the development of hydrophobic surfaces with hierarchal architectures^13^. For example, fabrics coated with dye incorporated silica nanoparticles were tested for the durability and wash fastness^14^, along with change in hydrophobicity with time^15^,^16^,^17^. Additionally, the degree of hydrophobicity to surface coating also reduced bacterial attachment leading to antimicrobial nanoparticles^18^.

Inorganic materials such as aluminum, zinc and titanium based metal oxides with NIR reflectance property and super hydrophobicity have been synthesized and reported in literature^19^,^20^. Organic fluorophores with high reflectance in the NIR region are rare and incorporation of these into a nanoparticle lattice offers many advantages such as low cost, biocompatiblity and easy accessibility. Consequently, the unique combination of hydrophobicity and NIR reflectance of such nanoparticles has led to the production of a diverse and multifunctional materials with interesting applications.

Herein, we explore the relationship between molecular organizations on the surface with the overall properties of the particle (Fig. 1). We report surface functionalization of silica nanoparticles with perylene derivatives and octadecyl groups to understand the significance of molecular arrangement on hydrophobic and optical properties of the particles. Three types of functionalised silica nanoparticles are used in this study, the first one involving particles with different concentrations of perylene molecules (**P-1**, **P-2 and P-3**), the second series with both perylene and octadecyl groups at different molar ratios (**PT-1**, **PT-2**) and the third one with only octadecyl groups (**T1**) on the surface. The questions addressed in this paper include, is it possible to control surface morphology using aggregation of perylene, which in turn influence the properties of the particles? Also, what would be the role of mixture of perylene and aliphatic long chain molecules towards the structure-property relationship of the nanoparticles? It is conceivable that flexible octadecylsilyl molecules fill the gaps between the perylene aggregates on the surface (**PT-1**, **PT-2**) and achieve complete coverage on the particle surface (Fig. 1).

## Results

### Synthetic Strategy and Characterization of Functionalised Silica Nanoparticles

**Figure 2:**
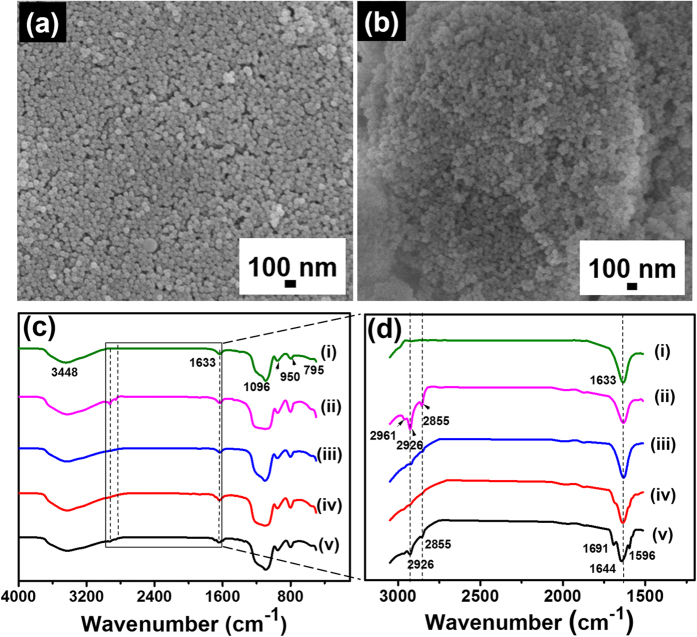
SEM image of monodispersed silica nanoparticles before (**a**) and after (**b**) functionalisation with perylene diimide molecules. FTIR spectra (**c**) of (i) SiO_2_ NPs, (ii) **T1** (TMODS), (iii) **P-1** (5 wt% PDI), (iv) **P-3** (21 wt% PDI), v) **PT-2** (1:1 molar ratio of PDI to TMODS) functionalised silica nanoparticles. The marked region in (**c**) is expanded and given in (**d**) to show corresponding peaks clearly. IR spectra of **P-2** and **PT-1** NPs are given in the Supplementary Fig. S1a.

Silica nanoparticles were synthesised using a reverse microemulsion method (see Supplementary Method S1) and fully characterised. Monodispersed silica nanoparticles were obtained with a narrow size distribution of around 40 nm (Fig. 2a).

PDI precursors were synthesised using a reported procedure^21^ (see Supplementary methods S2 and S3) and covalently attached to the surface of the silica nanoparticles. Different molar ratios of PDI and TMODS were functionalised onto the silica nanoparticle surface (Table 1). SEM images showed no difference in surface morphology before (pristine, Fig. 2a) and after functionalisation (P-3, Fig. 2b) on silica nanoparticles.

The FTIR spectra of the functionalised silica nanoparticles were recorded in the range of 500–4000 cm^−1^ in KBr matrix (Fig. 2c). Pristine silica nanoparticles were used as a control. The peaks observed at 3448 cm^−1^ and 1630 cm^−1^ corresponds to stretching vibrations of surface O-H groups of the silica nanoparticles. The peaks 1096 cm^−1^, 950 cm^−1^ and 795 cm^−1^ corresponds to Si-O-Si, Si-OH and Si-O bonds, respectively. Compared to pristine silica nanoparticles, octadecyl groups incorporated particles **PT-2** (1:1 molar ratio PDI to TMODS) and **T1** showed additional peaks at 2929 cm^−1^ and 2855 cm^−1^ which correspond to –CH stretching vibration of alkyl groups. The perylene incorporated particles (**P-1** to **P-3** and **PT-1**, **PT-2**) showed peaks at 1691–1644 cm^−1^ for imide C=O stretching and 1596 cm^−1^ for aromatic C = C stretching vibrations. These results suggest that the perylene and octadecyl moieties were successfully functionalised onto the surface of the silica nanoparticles.

**Figure 3:**
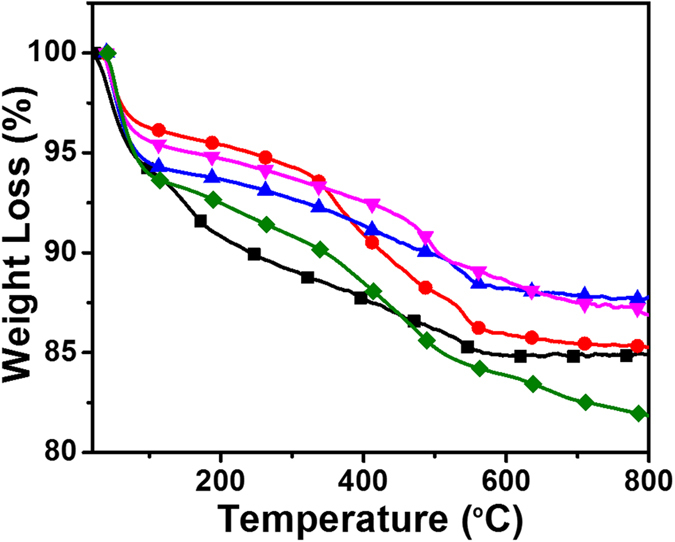
TGA traces of un-functionalised (◼) and functionalised (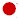) T1, (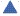) P-1, (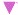) P-3, (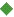) PT-2 silica nanoparticles. Please see TGA traces of P-2 and PT-1 NPs in the Supplementary Fig. S1b.

Thermogravimetric analyses of the functionalised silica nanoparticles were carried out under nitrogen atmosphere with a heating rate of 10 °C/min in the temperature range of 50–800 °C (Fig. 3) and the results are summarised in Supplementary Table S1. All TGA traces exhibit a three stage degradation process. The first step of degradation was observed below 150 °C corresponding to the loss of adsorbed water molecules. The second mass loss observed in the range of 150–400 °C, which can be assigned to the decomposition of organic groups and loosely bound Si-(OR) groups. The third mass loss observed in the range of 400–800 °C was due to degradation of both the organosilicate framework and the PDI groups on the surface of the particles^22^. TMODS functionalised (**T1**) and PDI functionalised **P-1**, **P-2** and **P-3** nanoparticles showed a 4%, 6%, 6% and 5% weight loss, respectively, below 150 °C as compared to the pristine silica nanoparticles (8%). The observed weight loss for the functionalised silica nanoparticles can be explained by the fact that the surface functionalization of silica nanoparticles reduces the adsorption of water. When the temperature was further increased from 150–800 °C, **T1** and **P-3** nanoparticles showed a 10.5% and 8.2% weight loss, respectively, which can be explained as the loss of organic moieties grafted on the silica surface. Further, **PT-1** and **PT-2** showed weight loss of 9% and ~11%, respectively. These results are also supported by the elemental analysis (see Supplementary Table S1).

### Photophysical Properties of PDI Functionalised Silica Nanoparticles

**Figure 4:**
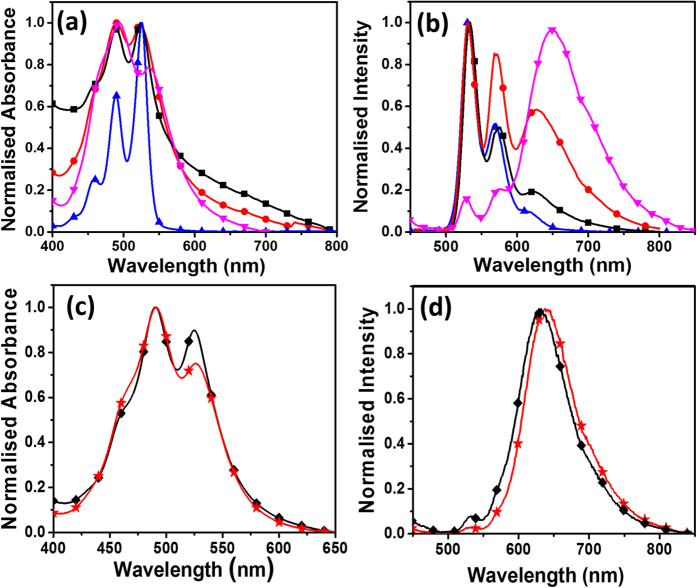
Normalised absorbance (**a**,**c**) and emission spectra (**b**,**d**) of (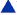) PDI silane precursor, (**◼**) **P-1** (5 wt% PDI), (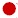) **P-2** (10 wt% PDI), (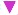) **P-3** (21 wt% PDI), (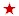) **PT-1** (1:0.01 molar ratio PDI to TMODS), (◆) **PT-2** (1:1 molar ratio PDI to TMODS) functionalised silica nanoparticles dispersed in THF. Excitation wavelength used was 350 nm.

The absorption and emission spectra of PDI and TMODS functionalised silica nanoparticles dispersed in THF are shown in Fig. 4.

The PDI silane precursors showed characteristic absorption peaks around 459, 489, and 526 nm corresponding to the S_0_–S_1_ transition with a well resolved vibronic structure from 0–0, 0–1, 0–2 and 0–3 transitions, respectively^23^. The absorption spectra of the PDI functionalized silica nanoparticles were broad with an inversion in peak intensities of 0–1 and 0–0 transition, compared to free PDI silane precursor in THF solution (Fig. 4a). The ratio of absorbance intensities of the 0–0 and 0–1 transitions are often used as an indication to determine the extent of aggregation^24^. The A_0-0_/A_0-1_ ratio from the absorption spectra of PDI silane precursor in THF was found to be ~1.6, which suggest the presence of free monomeric molecules in THF solution^25^. On functionalization of PDI onto the surface of the silica nanoparticles, the A_0-0_/A_0-1_ ratio was found to decrease with an increase in concentration of perylene on the surface and was 1.04 for **P-1** (5 wt% PDI), 0.99 for **P-2** (10 wt% PDI) and 0.79 (21 wt% PDI) for **P-3** nanoparticles. Strong vibronic coupling of H-aggregates results in an increase in intensity of the (0–1) vibronic band relative to the (0–0) transition band. Based on the absorbance intensity ratio, the PDI molecules on the surface of the **P-3** particles are more aggregated when compared to **P-2** and **P-1** particles. This is expected owing to the higher concentration of PDI molecules present on the surface. Introduction of PDI and TMODS in molar ratio of 1:0.01 (**PT-1**) and 1:1 (**PT-2**) led to changes in aggregation of the PDI molecules on the surface (Fig. 4c). The A_0-0/_A_0-1_ ratio from the absorption spectra of the **PT-2** was found to be 0.89, slightly higher than that for **PT-1** nanoparticles (0.75).

In order to understand the aggregation and arrangement of molecules on the surface, emission spectra of all particles were analyzed and compared with the solution spectra of PDI. The emission spectrum of PDI silane precursor in THF showed characteristic non-aggregated multiple maxima at 530, 568 and 618 nm (see Fig. 4b). Significant changes in the emission spectra of PDI molecules were observed after functionalization on the surface of silica nanoparticles (Fig. 4b). The nanoparticles with low concentration of PDI (**P-1**) showed an emission spectrum similar to the PDI silane precursor in solution. Upon increasing the concentration of PDI on the surface of the silica nanoparticles a new broad peak with a maximum around 650 nm was observed from **P-3** nanoparticles, which is similar to aggregated PDI dyes in solution (650 nm)^26^. This suggests that the variations observed in the emission maxima of particles with different PDI concentrations is related to aggregation of molecules on the surfaces, which then allows fine tuning of the color of emission from yellowish green to bright pink (see Supplementary Fig. S2). The emission properties of the PDI on the surface of silica nanoparticles arise from the formation of distorted H-aggregates with an increase in concentration^27^.

Interestingly, the **PT-1** (λ_emiss_ = 638 nm, Δ_P-3-PT-1_ = 12 nm) and **PT-2** (λ_emiss_ = 630 nm, Δ_P-3-PT-2_ = 20 nm,) particles functionalized with both PDI and alkyl groups showed blue shift in emission peaks with respect to P-3 particles (λ_emiss_ = 650 nm) indicating higher distortion of H-aggregates caused by the presence of long octadecylsilane groups (TMODS) on the surface as shown in Fig. 4d.

### Characterization of Superhydrophobic properties

**Figure 5:**
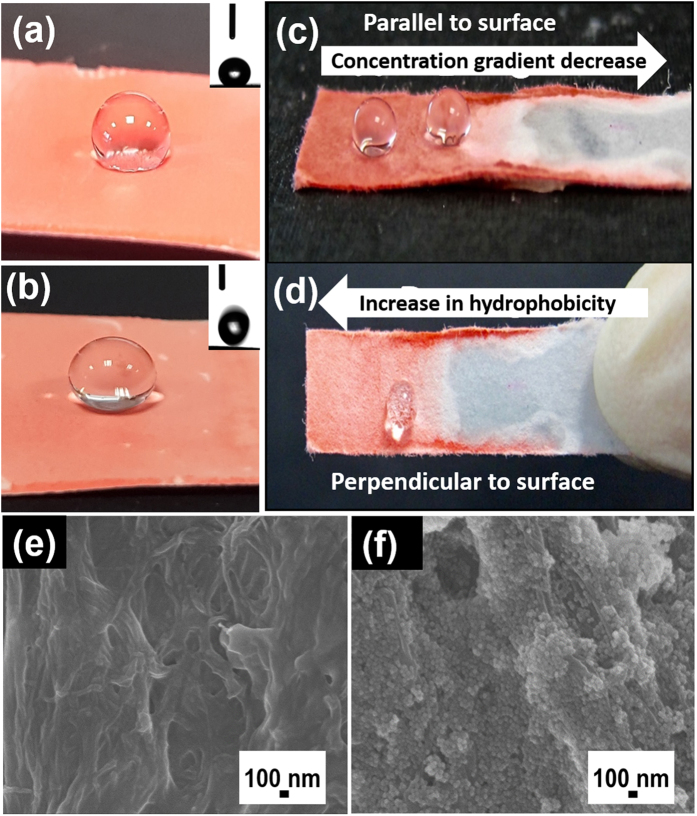
Image of water drop on the surface of (**a**) **P-3**, (**b**) **PT-2** coated silica TLC plate. Inset shows corresponding image of water drop taken during contact angle measurements, where drop does not stick to the surface coated with **PT-2** NPs. Effect of concentration gradient of **PT-2** (1:1 molar ratio PDI to TMODS) silica nanoparticles coated on filter paper held parallel (**c**) and perpendicular (**d**) to the surface in the presence of water droplet. SEM images of paper before (**e**) and after (**f**) coating with **PT-2** silica nanoparticles.

The wetting ability of surfaces coated with functionalised silica nanoparticles can be visualised by using highly polar substrates such as filter paper or silica-gel TLC plate (Fig. 5). The choice of the commercially available TLC plate was made to represent a highly hydrophilic rough surface. As expected, when the water droplet was placed on the unmodified commercially available silica TLC plate, complete absorption and spreading on the surface was observed indicating a superhydrophilic surface. In contrast, the **P-3** coated TLC plate surface was hydrophobic (CA~137.2°, Fig. 5a) and the **PT-2** coated surface was superhydrophobic and repelled the water droplet completely (CA~156°, Fig. 5b).

In order to further demonstrate the superhydrophobic effect of silica nanoparticles, a concentration gradient dependent hydrophobicity model was developed. One end of a long piece of filter paper of known length was dipped in THF solution of the dispersed **PT-2** NPs to achieve a concentration gradient effect across the filter paper. The concentration gradient was formed through a combination of capillary force mediated wetting of the paper and movement of solution upward from the solution front, along with continued evaporation of the solvent THF from the surface of the paper. After drying at room temperature, the hydrophobicity was measured using water drop test method (Fig. 5c).

It was observed that lower part of the filter paper exhibited superhydrophobic character, where the water droplet completely rolled off from the surface of the coated filter paper when tilted (Fig. 5d). But other regions, where concentration of the nanoparticles are low, water droplet stayed on the surface even when the paper was tilted owing to the low concentration of the nanoparticles and strong interaction of water molecules with the paper surface. Finally, at the region where the surface is completely hydrophilic, water is absorbed upon contact. The changes in hydrophobicity and the wettability with water is directly proportional to the presence of **PT-2** NPs on the surface of paper.

Superhydrophobicity depends on the surface energy and surface roughness of the sample. SEM images of the paper after coating of nanosilica (**PT-2**) showed uniform distribution of **PT-2** nanoparticles on the surface of the paper (Fig. 5f), when compared to pristine filter paper (Fig. 5e). In general, superhydrophobicity is significantly enhanced when the hydrophobic surface has a hierarchal structure with a combination of both nanometre and micrometre-sized roughness^28^.

**Figure 6:**
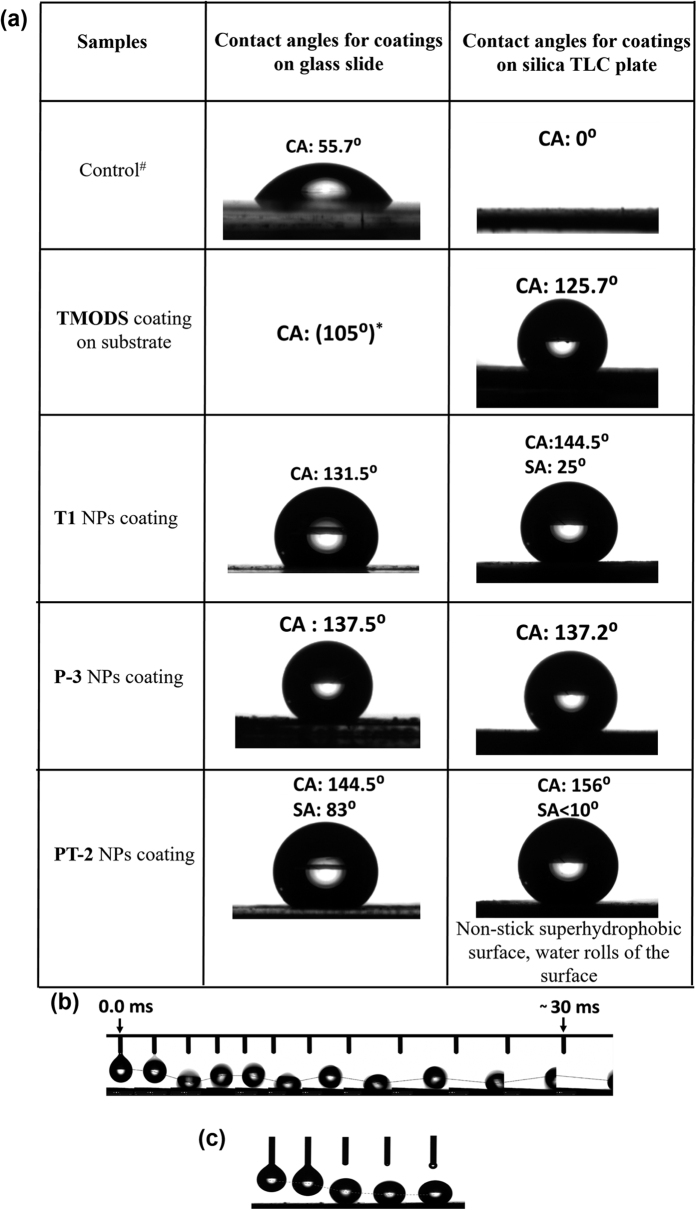
(**a**) Contact angle and sliding angles (CA and SA) of glass slide and silica TLC plate substrates coated with functionalised silica nanoparticles (^**#**^**Control** samples are bare glass cover slips and silica TLC plate, **P-3**, **PT-2**, functionalised silica nanoparticles, **T1** refers to TMODS functionalised silica nanoparticles. *As per ref. 34). Selected stills showing the anisotropic bouncing of a water droplet on a **PT-2** (**b**) and **T1** (**c**) coated TLC plate in a 30 ms time frame.

Contact angle measurements were performed using two types of substrates, namely, cleaned glass cover slip and silica TLC plate using goniometer measurements and summarised in Fig. 6a. Changes in contact angle were observed on glass surface when coated with functionalised silica nanoparticles. Bare clean glass surface provided a contact angle of only ~55.7°, and coating with octadecyl silanes increased the contact angle to ~105°. Coating with **PT-2** NPs led to the formation of superhydrophobic surface with a water contact angle of >150°. The increase in contact angle can be attributed to the formation of rough micro-nanomorphological pattern on these surfaces and reduction in surface energy of the particles.

Also, values of contact angle measured for the nanoparticle coated TLC plates were higher than the coated glass substrates. SEM micrographs of TLC plate showed random distribution of silica clumps of size around 8–22 μm with random pores (see Supplementary Fig. S3a) and coating of the functionalized silica nanoparticles led to a relatively less porous surface (see Supplementary Fig. S3b). As expected, commercially available TLC plate is superhydrophilic with complete absorption or spreading of water with a contact angle (CA) of ~0°. Upon coating with functionalised silica nanoparticles, an increase in the contact angle value was observed similar to particles coated on paper. **T1** particles showed a contact angle (CA) of 144° and a sliding angle of 25°. Similarly, PDI functionalised silica NPs (**P-3**) coated TLC plate also showed a CA of ~137°. Thus **PT-2** coated TLC plates showed different behaviour as compared to **T1** and **P-3** coated TLC plates. This could be due to the arrangements of molecules on the surface of silica particles **T1** and **P-3**, which could trap water droplets and thus reduces the contact angle (See Supplementary Fig. S4). **PT-2** coated TLC plates exhibited non-sticky, water repellent properties with CA of ~156° and sliding contact angle (SA) less than 10° indicating superhydrophobicity.

Video footage taken using a high speed camera was used to further demonstrate the water repellent properties of the nanoparticle coated surface. The kinetic energy of the droplet was transformed into vibrational energy on touching the **PT-2** coated surface leading to the anisotropic bouncing of the water droplet without allowing it to rest in the Cassie state (Fig. 6b)^29^. As a control, the same experiment was performed with a **T-1** (TMODS) coated surface (Fig. 6c). In contrast to the **PT-2** system, when the water droplet fell on the **T-1** coated surface it did not rebound from the surface.

**Figure 7:**
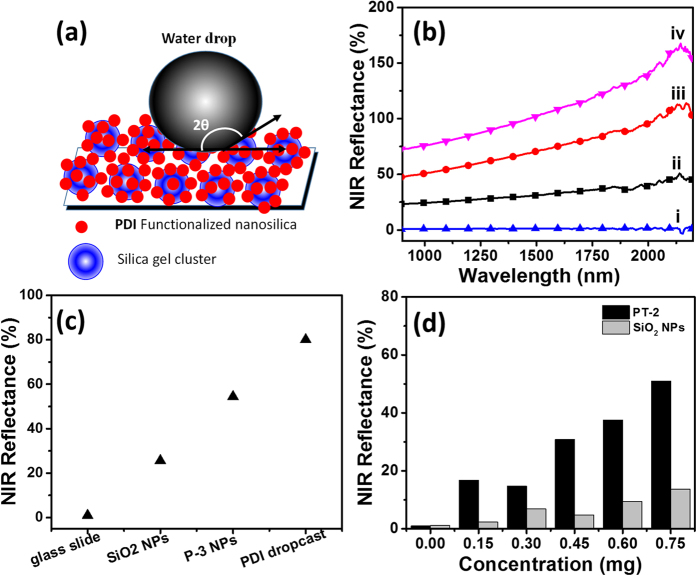
(**a**) Cartoonistic representation of water droplet on the surface of the **PT-2** nanoparticle coated TLC surface. (Objects are not in scale). (**b**) % NIR reflectance of (**i**) glass cover slip, (**ii**) unfunctionalised silica nanoparticles, (**iii**) **P-3** (21 wt% PDI), (**iv**) perylene diimide (PDI) drop cast on a glass cover slip in the range of 800–2200 nm. (**c**) The plot of (%) NIR reflectance of different materials and (**d**) different concentrations of SiO_2_ and **PT-2** (1:1 molar ratio PDI to TMODS) nanoparticles drop casted on glass cover slip.

The wettability of a material is often attributed to a low surface free energy and a high degree of roughness^30^. The ‘Lotus effect’ explains how hierarchal structures with micro- and nanoscale roughness can lead to superhydrophobicity and an extreme non-stick and water-repellent surface^28^. Higher magnification SEM images (see Supplementary Fig. S3b) of both **P-3** and **PT-2** coated TLC plates showed similar distribution of silica nanoparticles on the surface of TLC plate. Thus, hydrophobicity observed for all functionalised silica NPs (**T1**, **P-3** and **PT-2**) coated TLC plates is due to micro-nanoroughness achieved by the assembly of functionalised silica nanoparticles on silica clusters. The distribution of nanoparticles may increase the amount of trapped air in between the particles, which then enhances the hydrophobicity, as described in the Cassie-Baxter model^31^. In addition, micro-nanoscale roughness was formed by the assembly of multiple layers of nanoparticles (Fig. 7a). Similar to the lotus leaf, the **PT-2** coated surface possess a hierarchical structure from multiple layers of surface functionalised silica nanoparticles with hydrophobic groups (i.e. PDI and TMODS).

### NIR reflectance of PDI functionalised nanoparticles

Approximately 52% of ultraviolet radiation that reaches the Earth is in the near infrared region (700–2300 nm) of the electromagnetic spectrum. Absorption of radiation in this region will eventually lead to heat gain in many objects^32^. As a counter measure, NIR reflective colorants are used as an effective means of reflecting such radiations. Since perylene derivatives have shown NIR reflectance^8^,^10^, it is conceivable that PDI functionalised silica nanoparticles will also exhibit such properties.

The diffuse reflectance results obtained from PDI functionalised silica nanoparticles (**P-3**) coated on glass substrate using Teflon as a reference is shown in Fig. 7b. A narrow 1100 nm wavelength radiation was chosen for our experiment^19^. From the data obtained (Fig. 7c), PDI functionalised silica nanoparticles showed NIR reflectance up to 52% compared to pristine silica nanoparticles (28%). Also, glass substrate coated with PDI showed maximum reflectance (80%) in accordance with previous literature^8^,^10^. Such increase in NIR reflective properties after functionalisation of nanoparticle with PDI could be used to reduce the build-up of heat in materials from the absorption of NIR radiation.

The concentration dependent NIR reflectance studies were carried out on **PT-2** silica nanoparticles and compared to pristine silica nanoparticles coated on a glass substrate as a control (Fig. 7d). The maximum reflectance (50%) was obtained from **PT-2 NPs** compared to bare silica nanoparticles showing only 15% reflectance for the same amount (0.75 mg) dispersed on glass cover slip. Further optimization of experimental variables such as the size of nanoparticles, concentration of PDI on the surface, coating processes, is currently in progress to optimize the NIR reflective properties. From the preliminary experiment, it is noted that PDI functionalised on silica nanoparticles do exhibit NIR reflective property.

## Discussion

Six functionalised silica nanoparticles were synthesised, fully characterised and properties were investigated. The absorption and emission spectra of the samples showed correlation with the amount of perylene diimide (PDI) groups incorporated on the surface of the silica nanoparticles. Increasing the concentration of PDI, for example, led to a red shift in absorption maxima, changes in peak intensities and loss of vibrational fine structure demonstrating the aggregation induced formation of excimeric state. By using a mixture of PDI and TMODS on silica nanoparticle surface, the aggregation of PDI and organic molecule coverage on the particle surface were modulated. Among the six types of nanoparticles prepared, **PT-2** showed the maximum coverage and fascinating properties. Pristine silica nanoparticles were hydrophilic (CA = 0°) and PDI functionalised nanoparticles showed hydrophobicity (CA = 137.2°). However, co-immobilization of the PDI and TMODS on the surface of particles led to superhydrophobic nanoparticles (**PT-2**) with a CA of >150° and a sliding contact angle (SA < 10°). The high hydrophobicity of the **PT-2** is explained based on the effective coverage of the surface with organic molecules. Furthermore, the high NIR reflectance of PDI derivatives was used towards developing silica nanoparticle coatings with more than 80% NIR reflectance. In conclusion, here we report the successful synthesis of a fluorescent, superhydrophobic, self-cleaning NIR-reflective silica nanoparticles for coating on sensitive instruments.

## Methods

### Materials

Tetraethylorthosilicate (TEOS), (3-aminopropyl)triethoxysilane (APTES), perylene-3,4,9,10-tetracarboxydianhydride and trimethoxy(octadecyl)silane (TMODS) were obtained from Sigma Aldrich. Aqueous ammonia solution (25%) was obtained from Merck. All chemicals and solvents used were AR grade and used without further purification.

### Characterization Methods

Nuclear magnetic resonance (^1^H NMR) spectra were recorded on Bruker Avance AV300 (300 MHz) NMR instrument using CDCl_3_ as the solvent. Absorption and emission spectra were measured on a UV-1601PC Shimadzu spectrophotometer and RF-5301PC Shimadzu spectrofluorophotometer. Bruker ALPHA FT-IR spectrophotometer was used for records Fourier transform infrared (FT-IR) spectroscopy analysis. Thermogravimetric analyses (TGA) were conducted using a SDT 2960 TA instrument. All samples were heated under nitrogen atmosphere from 25 °C to 800 °C using a heating rate of 10 °C/min. FT-IR spectra were recorded in the range of 3800–400 cm^−1^ using a Varian Excalibur 3100. Samples were mixed with KBr powder and grounded in an agate mortar before being pressed into a disk for recording the spectrum.

### Contact Angle Measurement

The macroscale contact angle measurements were carried out using the video-based fully automated data physics optical contact angle microlitre dosing system (OCA 40 Micro, Germany). Deionised water drops (1 μl/drop) with known surface tension were dispensed using Teflon coated motor driven syringe. The contact angles were measured at be measured at 22 °C and 45% RH conditions and a video was recorded (72 frames/second) for every dispensed solvent droplet. Any dynamic changes to the droplet on the surface could be precisely observed through this method^33^.

Functionalised silica nanoparticles (0.005 g) were dispersed in THF (2 ml) and sonicated for 10 minutes. TLC plates were dip coated in the dispersion of silica nanoparticles in THF and left to dry at room temperature. This procedure was repeated several times until a uniform coating was achieved.

For coating on glass cover slips, aqueous NaOH solution (10%) was used to clean the surface and dried prior to drop casting the solution of silica nanoparticles in THF.

Finally, a gradient coating of functionalized silica nanoparticles on a strip of filter paper (0.5 × 1.0 cm) was achieved by touching one end of the paper to the nanoparticle solution and letting the capillary action draw the solution to the paper. During the rise of solution through the paper, the solvent THF tends to evaporate and deposit the particles on the paper. This process allowed us to create a gradient with more nanoparticles at the end of the paper closer to the solution and less particles at the other end. Since the nanoparticles are red in color, the capillary gradient coating is visible with the naked eye. Three sections with high, medium and low concentrations of nanoparticles on coated filter paper were then tested to demonstrate how the degree of hydrophobicity was dependent on concentration of the particles adsorbed.

### NIR Reflectance Measurement

The NIR diffuse reflectance of the silica nanoparticles coated glass substrate was measured using UV-Vis-NIR spectrophotometer (Shimadzu, UV-3600) with an integrating sphere attachment, ISR-3100. The NIR reflectance measurements of the samples were performed within the wavelength range of 700–2200 nm.

### General Procedure for Functionalization of Silica Nanoparticles

Silica nanoparticles (100 mg) dispersed in dry toluene (10 ml) under nitrogen atmosphere were treated with PDI silane precursors and/or TMODS at different ratios to functionalise the surface (see Table 1). The mixture was refluxed under constant stirring at 90 °C for 24 hours, centrifuged to obtain the solid and washed with THF to remove unreacted silane precursors before drying.

A summary of the amounts of reagents used for the synthesis of functionalised nanoparticles is given in Table 1, where **P-1**, **P-2** and **P-3** refers to 5, 10 and 21 wt% of PDI, respectively, in solution, and **PT-1** and **PT-2** refers to a 1:0.01 and a 1:1 molar ratio of PDI to TMODS reagents, respectively, and **T1** refers to silica nanoparticles functionalized with TMODS only.

## Additional Information

**How to cite this article**: Sriramulu, D. *et al.* Synthesis and Characterization of Superhydrophobic, Self-cleaning NIR-reflective Silica Nanoparticles. *Sci. Rep.* **6**, 35993; doi: 10.1038/srep35993 (2016).

**Publisher’s note:** Springer Nature remains neutral with regard to jurisdictional claims in published maps and institutional affiliations.

## Supplementary Material

Supplementary Information

## Figures and Tables

**Figure 1:**
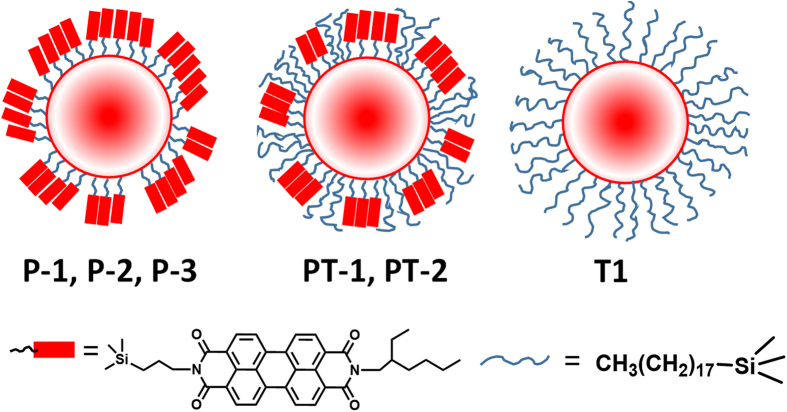
Cross sectional view of the conceptualized structures of the three silica nanoparticles prepared during this study. Full composition of each of these nanoparticle is given in the experimental section.

**Table 1:** Amount of reagents used for the synthesis of different batches of functionalized nanoparticles and the intensity ratios for the first and the second absorption peaks of perylene silica nanoparticles as compared to reference PDI silane precursor dissolved in THF.

Sample	PDI (mg)	TMODS (μL)	% Ratio	λ_abs_ (nm)	λ_emi_ (nm)	Absorbance ratio	TGA (%wt. loss)
P-1	5.0	0	5	523	534	1.04	6%
P-2	10.0	0	10	490	530, 628	0.99	5%
P-3	21.0	0	21	494	650	0.78	8.2%
PT-1	18.0	0.13	1:0.01	491	638	0.75	9%
PT-2	18.0	12.8	1:1	490	630	0.89	11.3%
T1	—	18.0	18	—	—	—	10.5%
PDI silane precursor	—	—	—	526	530	1.6*	—

(*) values from solution measurement, for all cases, 100 mg of silica particles were used to start the reaction.
