# Supplementary material for: Synthesis and Characterization of Superhydrophobic, Self-cleaning NIR-reflective Silica Nanoparticles
Source: Sci Rep. 2016 Nov 8;6:35993. doi: 10.1038/srep35993 (PMC5099900; doi:10.1038/srep35993)
Supplement: Supplementary Information [file srep35993-s1.pdf]

Supporting Information for the manuscript

Synthesis and Characterization of Superhydrophobic, Self-cleaning NIR-reflective Silica Nanoparticles

Deepa Sriramulu[1], Ella Louise Reed[1], Meenakshi Annamalai[2], Thirumalai Venky Venkatesan[2,3,4,5,6], Suresh Valiyaveettil[1]*

[1]Department of Chemistry, 3 Science Drive 3, National University of Singapore (NUS), Singapore 117543.
[2]NUSNNI-NanoCore, National University of Singapore 117411, Singapore
[3]Department of Physics, National University of Singapore 117542, Singapore
[4]Department of Electrical and Computer Engineering, National University of Singapore 117576, Singapore
[5]Department of Material Science and Engineering, National University of Singapore 117575, Singapore and
[6]NUS Graduate School for Integrative Sciences & Engineering, National University of Singapore 117456, Singapore
Email: *chmsv@nus.edu.sg

Supplementary method S1. Synthesis of Silica Nanoparticles via the Reverse Microemulsion Method[1]

Cyclohexane (120 ml) was added to Triton–X (26.4 ml) and octanol (25.2 ml) and left to stabilise for 5 minutes. Deionised water (8 ml) was added dropwise to the mixture and stirred for 10 minutes. TEOS (10 mmol, 2.4 ml) and aqueous ammonia solution (0.13 mol, 2.4 ml) together was then added. The reaction mixture was stirred for 24 hr and excess acetone was added to break the emulsion. The mixture was centrifuged and washed with ethanol and water. The nanoparticles obtained were then left to dry in a vacuum oven overnight at 72 °C.

Supplementary method S2. Preparation of N-(2-ethylhexyl)perylene-3,4-anhydride-9,10-imide.[2]

Perylene-3,4,9,10-tetracarboxydianhydride (1 mmol, 0.4 g) and alkylamine solution (8 mmol, 1.0 g) were added to a 1:1 mixture of ethanol and water (50 ml), and refluxed for 6 hours at 90 °C. After completion, the reaction was cooled to room temperature and acidified with hydrochloric acid (10 %). The precipitate was filtered and washed with water to remove any unreacted amine, dissolved in hot potassium hydroxide solution (1%) and centrifuged to remove the insoluble diimide precipitate. After centrifugation, the filtrate was filtered again to remove the diimide completely. To the filtrate, potassium chloride solution (10 %) was added and filtered. The obtained solid was dispersed in water and acidified with hydrochloric acid (10 %). The red precipitate formed was filtered and washed with water. ^{1}H NMR (300 MHz, $CDCl_3$, δ ppm): 8.75-8.65 (8H, m), 4.2 (2H, t), 1.93 (1H, m), 1.34 (8H, m), 0.98 (6H, m). MS (APCI-TOF): calculated for $C_{32}H_{25}NO_5$: 503.1738, found: 503.1714.

Supplementary method S3. Synthesis of N-(2-ethylhexyl)-N'-(3-(triethoxysilyl)propyl)perylene-3,4,9,10-tetracarboxylic acid diimide precursor.[3]

Perylene monoimide (PMI, 0.06 mmol, 0.03 g) was mixed with APTES (4 mmol, 0.9 g) and refluxed under argon atmosphere for 5 hours under constant stirring. After cooling to room temperature, the mixture was washed with hexane to remove any unreacted amines. The red solid product was dried under reduced pressure and stored in nitrogen atmosphere. . ^{1}H NMR (300 MHz, $CDCl_3$, δ ppm): 8.62-8.49 (8H, m), 4.22-4.12 (2H, m), 3.87-3.80 (6H, m), 3.75-3.68 (2H, m), 1.98-1.85 (5H, m), 1.58-0.82 (23H, m). MS (APCI-TOF): Calculated for $C_{41}H_{46}N_2O_7Si$: 706.3074, found 707.3131 $(M+H)^+$

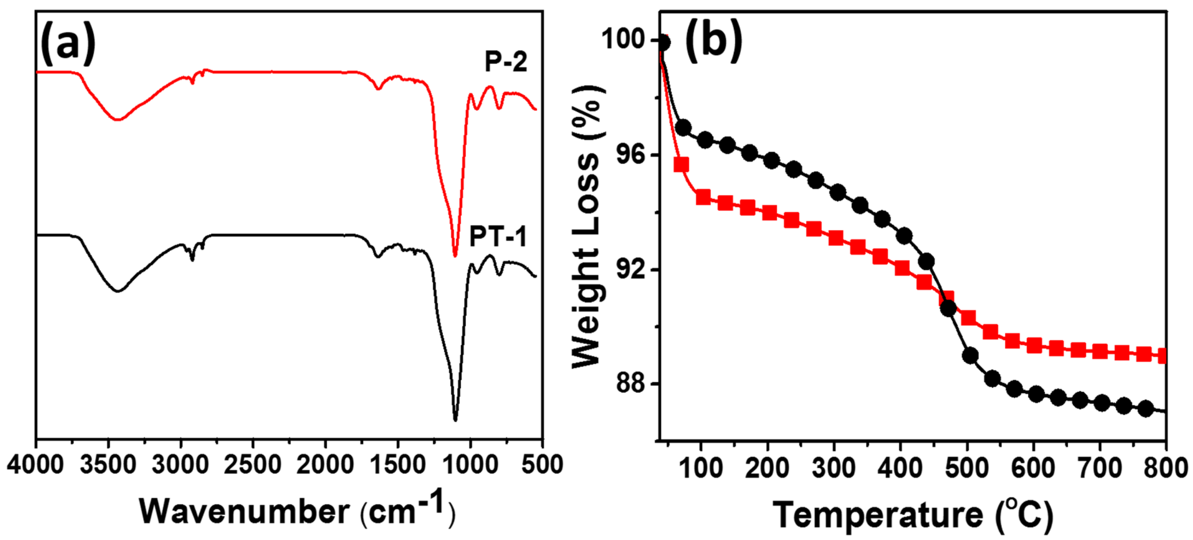

Supplementary Figure S1. FTIR spectra (a) and TGA traces (b) of **P-2** (■), **PT-1** (●) functionalised silica nanoparticles.

Supplemenatry Table S1. Elemental analysis of functionalised silica nanoparticles samples

Sample	C%	H%	N%	< 150° C	150° - 800° C	400° - 800° C
**P-1**	1.71	1.85	< 0.50	6%	6 %	3.5%
**P-2**	2.11	1.52	< 0.50	6%	5%	3%
**P-3**	6.98	1.84	< 0.50	5%	8.2%	5.69%
**PT-1**	5.89	2.16	< 0.50	3.8%	9%	6%
**PT-2**	7.42	2.48	< 0.50	6.8%	11.3%	6.6%
**T1**	5.49	2.35	< 0.50	4.3%	10.5%	6%

P-1 (5 wt % PDI), **P-2** (10 wt % PDI), **P-3** (21 wt % PDI), **PT-1** (1:0.01 molar ratio PDI to TMODS), **PT-2** (1:1 molar ratio PDI to TMODS), **T1** (TMODS) functionalized silica nanoparticles.

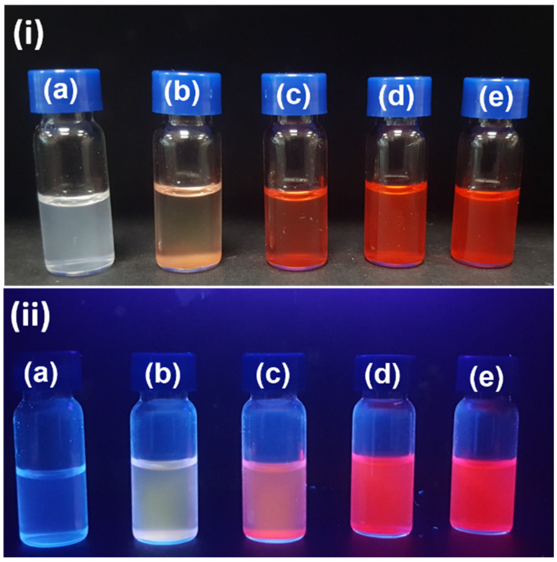

Supplementary Figure S2. Optical images of **(a)** $\mathbf{SiO_2}$ **NPs**, **(b) P-1** (5 wt % PDI), **(c) P-2** (10 wt % PDI), **(d) P-3** (21 wt % PDI), **(e) PT-2** (1:1 molar ratio PDI to TMODS) functionalised silica nanoparticles dispersed in THF (5 mg / ml). Illuminated under **(i)** white light and **(ii)** UV light (365 nm).

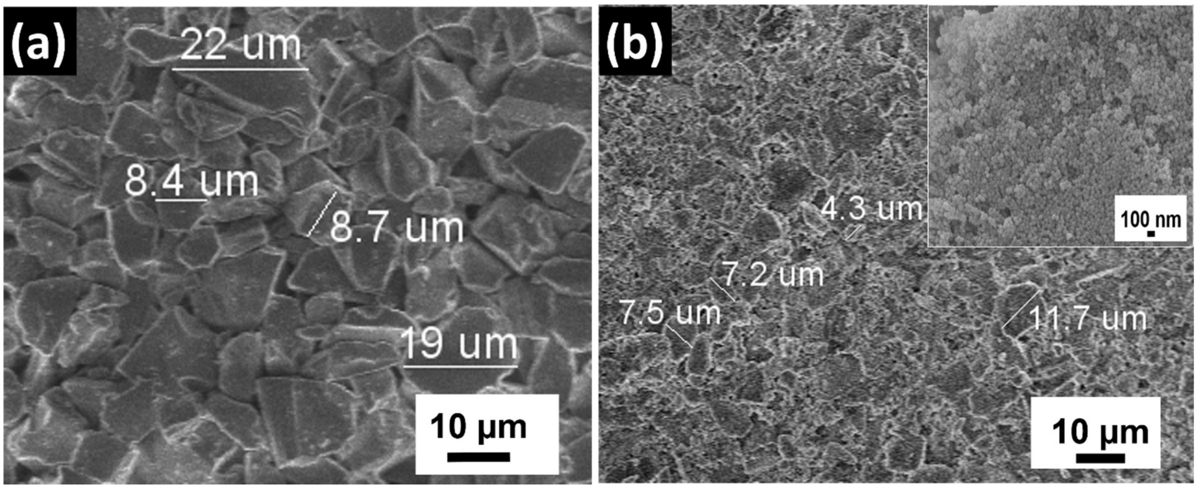

Supplemenatry Figure S3. SEM images of silica TLC plate before (**a**) and after (**b**) coating **PT-2** NPs coating, inset is the corresponding high magnification image of **PT-2** NPs on silica TLC plate.

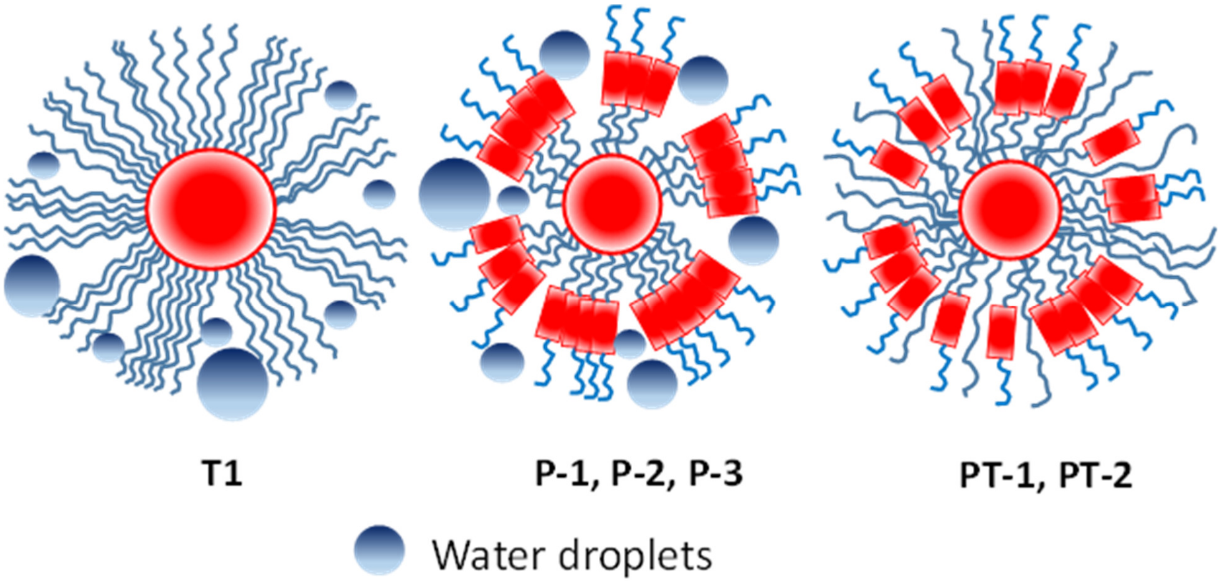

Supplementary Figure S4: Cartoonistic representation of the interaction of functionalised silica particles with water.

References

1. Bagwe, R. P., Yang, C., Hilliard, L. R. & Tan, W. Optimization of Dye-Doped Silica Nanoparticles Prepared Using a Reverse Microemulsion Method. *Langmuir* **20**, 8336-8342 (2004).

2. Huang, H., Che, Y. & Zang, L. Direct synthesis of highly pure perylene tetracarboxylic monoimide. *Tetrahedron Lett.* ***51***, 6651-6653 (2010).

3. Ribeiro, T., Baleizão, C. & Farinha, J. P. S. Synthesis and characterization of perylenediimide labeled core-shell hybrid silica-polymer nanoparticles. *J. Phys. Chem. C*. **113**, 18082-18090 (2009).
